# Experiences of Wheelchair Users With Spinal Cord Injury With Self-Tracking and Commercial Self-Tracking Technology (“In Our World, Calories Are Very Important”): Qualitative Interview Study

**DOI:** 10.2196/65207

**Published:** 2025-04-15

**Authors:** Vasiliki Mylonopoulou, Katerina Cerna, Alexandra Weilenmann, Mattias Rost, Tobias Holmlund

**Affiliations:** 1 Applied IT University of Gothenburg Gothenburg Sweden; 2 School of Information Technology Halmstad University Halmstad Sweden; 3 Neurobiology, Care Sciences and Society Karolinska Institute Stockholm Sweden

**Keywords:** wheelchair, spinal cord injury, tracking, self-tracking, wellness technology, calories, health inequalities, inclusive design in mobile health, design, lifestyle app, artificial intelligence, AI

## Abstract

**Background:**

Commercial wearable and mobile wellness apps and devices have become increasingly affordable and ubiquitous. One of their aims is to assist the individual wearing them in adopting a healthier lifestyle through tracking and visualizing their data. Some of these devices and apps have a wheelchair mode that indicates that they are designed for different types of bodies (eg, wheelchair users with spinal cord injury [SCI]). However, research focuses mainly on designing and developing new condition-specific self-tracking technology, whereas the experiences of wheelchair users with SCI using self-tracking technology remain underexplored.

**Objective:**

The objectives of this study were to (1) provide a comprehensive overview of the literature in the field of self-tracking technology and wheelchair users (as a basis for the study), (2) present the self-tracking needs of wheelchair users with SCI, and (3) present their experiences and use of commercial self-tracking technology.

**Methods:**

We conducted semistructured interviews with wheelchair users with SCI to understand their experiences with self-tracking and self-tracking technologies, their self-tracking needs, and how they changed before and after the injury. The interviews were thematically analyzed using an inductive approach.

**Results:**

Our findings comprised three themes: (1) being a wheelchair user with SCI, (2) reasons for self-tracking, and (3) experiences with self-tracking technologies and tools. The last theme comprised 3 subthemes: self-tracking technology use, trust in self-tracking technology, and calorie tracking.

**Conclusions:**

In the Discussion section, we present how our findings relate to the literature and discuss the lack of trust in commercial self-tracking technologies regarding calorie tracking, as well as the role of wheelchair users with SCI in the design of commercial self-tracking technology.

## Introduction

### Background

Every year, up to 500,000 people experience spinal cord injury (SCI) and, as a result, become wheelchair users [1]. This population must adjust to their new body, learn to use a wheelchair, and sustain an active lifestyle. An active lifestyle is vital for their independent living and quality of life. If they are not active, their shoulders can be injured because they are overused in their daily life without being trained for specific activities (eg, getting in and out of the wheelchair). Another risk is that they can gain weight, which requires them to change to a wider wheelchair, thus rendering more places inaccessible.

As commercial self-tracking technology (eg, smartwatches) becomes increasingly common, wheelchair users can access and benefit from its advancements. Some of this technology (eg, Apple Watch and Runkeeper) has already implemented accessibility settings for wheelchair users. Up to now, research on self-tracking technology has focused on (1) chair-ables, artifacts placed on or in the wheelchair to promote a healthier lifestyle [2-4]; and (2) studies on wheelchair users’ experiences with self-tracking technology that was provided or introduced to them [5-7]. However, the everyday lived experience of self-tracking and related technology (ie, self-tracking technology) of wheelchair users with SCI is underresearched. This can result in the exclusion and underrepresentation of this specific group in the research and development of commercial wearables. Excluding part of the population as “special users” is an ethical and social issue [8,9]. In addition, more commercial technologies are anticipated to become accessible to a broader audience once the European Accessibility Act (EAA) is applied. This act focuses on digital products and services, underlining the importance of mainstream technology being accessible to people with disabilities and older adults [10].

The objective of this study is to build on previous research on self-tracking and self-tracking technology among wheelchair users with SCI [5-7,11] to include lived experience of self-tracking and self-tracking technology. Through an interview study, we aimed to answer the following question: How do wheelchair users with SCI experience self-tracking and commercial self-tracking technology in their everyday lives?

This section first provides an overview of SCI and the impact of the injury on people’s lives. This is followed by a short overview of existing research on wheelchair users and self-tracking technology for health and well-being. Finally, we position our research in the context of contemporary society and technological research focusing on the role of wheelchair users with SCI.

### SCI Overview

Worldwide, approximately 90 million people live with SCI, many of whom are either underage or part of the working population [1,12]. Each year, between 250,000 and 500,000 people sustain an injury to their spinal cord [1]. The average lifetime costs of an SCI starting at the age of 25 years range from €0.45 million to €2.1 million (US $0.49 million-$2.3 million), which exceeds those related to dementia, multiple sclerosis, and cerebral palsy [1]. Poor quality of life for people with SCI, their families, and caregivers—in combination with the socioeconomic burdens caused by the injuries—make it imperative to find solutions for this condition.

A motor-complete SCI leads to complete muscle mass loss in the legs and, thus, life as a wheelchair user. For a walking person, the muscles in the legs are the main consumers of energy both in an active state (eg, when the person exercises or moves) and in a rest state (eg, sleeping or sitting) [13,14]. In addition, an injury above thoracic level 6 provokes an altered (physiological) autonomic response during both rest and activity [15]. This results in an altered metabolic response, leading to reduced energy burn in both states [13-15]. Body weight balance is partly explained as caloric balance based on calorie intake and output [16-18]. After an SCI, the total energy burn (ie, calorie burn) is much lower than before. Generally, after the initial acute phase, people with SCI lose weight due to the loss of muscle mass and the healing process. During rehabilitation, most people get used to their light body weight and narrow wheelchair, facilitating an active and independent daily life. Throughout the rehabilitation phase, body weight tends to increase (approximately ≥2 kg during the first year) [19]. This relates to the lack of compensatory caloric intake due to the lower metabolic demands (total daily energy burn), which leads to energy imbalance with higher calorie intake than energy burn [19]. Increased body weight can have many consequences, such as higher stress on shoulder muscles due to extra weight being carried each time the person transfers to and from their wheelchair [20]. Moreover, it becomes more difficult to manage daily activities such as self-care. The resulting shoulder pain and reduced independence are often devastating [20]. Thus, calories are extremely important for people with SCI. Maintaining an appropriate calorie balance becomes much more achievable when energy burn and calorie intake can be monitored.

### Wheelchair Users With SCI and Self-Tracking Technology for Health and Well-Being

#### Overview

Self-tracking is a voluntary, reflexive practice in which people collect data about themselves to enhance self-awareness and understanding by observing, noting, and interpreting various aspects of their lives [21]. Self-tracking technology is any technology that supports people in self-tracking practice. This technology can include apps—where the user registers information about their health and well-being—smartwatches or other wearable technology (wearables), or sensors embedded in assistive technology such as a wheelchair (chair-ables). Self-tracking technology is not assistive technology as it does not aim to improve the functional ability of people with disabilities or enable and enhance their participation and inclusion in different domains of life [22]. However, self-tracking technologies can offer inclusivity through adjusting functionality to include people with disabilities.

In this study, we focused on the experiences of people with SCI with mainstream technology as we aimed to contribute to an inclusive design. However, we recognize the importance of understanding and learning from assistive technology for our target demographic. This understanding could direct inclusive design research and practice as it provides insights into adjusting or expanding the mainstream technology to be more inclusive.

#### Assistive Technology

Chair-ables have been researched in terms of tracking specific behaviors and self-care–related injuries. Let us take the case of pressure releases, which are vital for wheelchair users in avoiding pressure ulcers potentially contributing to premature death [23]. A chair-able can support wheelchair users with pressure release exercises. Sensors can be placed on the wheelchair to measure in-seat movement, classify the activity from the sensors into weight shifts, and inform the user with their data as well as sending push notifications upon achieving goals related to pressure releases [2]. In 2021, Ahad et al [4] developed and evaluated the reliability of an algorithm and a chair-able that could support people on wheelchairs in doing their pressure release exercises. The year after, design considerations for supporting electric wheelchair users with SCI on their pressure releases were published [24]. The authors [24] implemented context-aware and unobtrusive reminders delivered by the wheelchair for incomplete or incorrect pressure release exercises. The interaction with the wheelchair in this context [24] was preferable to an interaction with an app. Similarly, but in the context of self-tracking apps, Amann et al [25,26] used participatory design to design, develop, and evaluate an evidence-based app that uses a smart camera to prevent pressure injuries.

As for self-care, Büyüktür et al [3] presented requirements for semiautomated tracking to support people with SCI in their self-care by interviewing patients and health care professionals. Their main findings were that the patients’ routines change from the clinic to the home environment and each patient decides to focus on what they value the most (eg, sleeping instead of waking up to change position during their sleep). Each patient and their informal caregivers try different routines in the home environment until they find one through which they can track the behavior and with which they can comply. Thus, Büyüktür et al [3] recommended a highly tailored and flexible system that can follow the patient’s routines as they change and give them feedback. In general, a systematic literature review [11] showed that there is research on self-management of SCI in the home environment and clinic as well as the promotion of physical activity and a healthy lifestyle (eg, wellness and fitness). For example, one of the articles reviewed [27] presented that an intervention that tracked the activity of wheelchair users with SCI and motivated them through just-in-time messaging had the potential to improve physical activity for wheelchair users with SCI. Similarly, an interview study [28] explored the barriers that people with SCI face to staying physically active: (1) lack of tailored physical activity forums with wheelchair users with SCI and (2) lack of personalized fitness-tracking technology tailored to wheelchair-based activities.

Mo et al [29] explored the information needs of wheelchair users, and they identified a lack of tailored, accurate, and affordable tracking for wheelchair users both commercially and academically. Even though they argued for inclusive technology, the start of filling this gap could be in the assistive technology literature. Li et al [30] introduced WheelPoser, a system that tracks and identifies wheelchair users’ on-the-move activity. They argued that they could do that by using 4 small sensors. Their dataset, code, and models are available for everyone to use, which allows mainstream technology to become more inclusive by using the same system as an add-on to their trackers or only the dataset to train their own systems. Huang et al [31], recognizing the lack of data on wheelchair users for training artificial intelligence algorithms on pose estimation, developed and evaluated a dataset to fill that gap. Their dataset is publicly available as well. Another article published in a disability forum explored the relationship between the successful completion of activity guidelines for people with SCI and the fitness or health status of the person with SCI [32]. This paper referred to activity guidelines [33,34] for people with SCI and an understanding of how mainstream technology can tailor its features (eg, daily goals) to a specific SCI group.

#### Inclusive Technology

Research underlines the need for inclusive and accurate commercial technology for wheelchair users through studies [35] and literature reviews [36,37]. The participants in the study by Li et al [35] showed interest in tracking their activity, but they did not trust the accuracy of contemporary commercial technology, understanding that those using powered wheelchairs could track some data on their activity that were relevant to them, such as distance. A literature review explored how academic publications in the personal informatics field address the information needs of wheelchair users [36]; it consolidated recommendations to tackle the design challenges that researchers and practitioners may face when attempting to design for including wheelchair users’ activity tracking and information needs. In 2019, Moon et al [37] conducted a literature review of studies on the inclusivity of contemporary digital technology. They advocated for inclusive and universal design to become an integral part of commercial digital technology’s development process and consolidated methods that can support the process.

Another systematic literature review [11] published in 2023 examined the use of mobile health in supporting people with SCI to maintain or improve their health. It showed that research lacked results regarding the role of commercial self-tracking technology for health and wellness to support people with SCI. The authors [11] attributed this gap in research to the fact that commercial self-tracking devices are inaccurate in their calculations for wheelchair users. However, in 2021, a study [38] evaluated the accuracy of the Apple Watch Series 1, showing that the watch was accurate for high-frequency strokes on a wheelchair (eg, a treadmill) but not for low-frequency strokes (eg, walking). Even though the research was published recently, the results of this research may not correspond to the current version of the Apple Watch (Series 7).

Related to commercial trackers and their use by of wheelchair users with SCI or their preferences, there is research limited to commercial technology given to them for a specific amount of time. For example, Ungurean and Vatavu [7] published a study in 2022 exploring the needs of wheelchair users related to activity trackers regarding different self-tracking technologies (eg, rings, watches, armbands, and chair-ables). The researchers [7] interviewed participants after letting them watch videos of 2 different self-tracking technologies (eg, ring and armband) to understand their perceptions, preferences, and willingness to use wearables. Some of their participants were already wearable technology users, and the researchers mentioned that this could have supported them in answering the interview questions [7]. However, the study’s results focused on the videos that the participants watched.

Malu and Findlater [5] based their research on the work by Carrington et al [6]. Carrington et al [6] tracked variables of wheelchair basketball players and presented the variables to the athletes to understand their needs and potential data use. In addition, Malu and Findlater [5] included wheelchair users who were not necessarily athletes. Their participants were interviewed, participated in design workshops, and tried 2 commercial technologies. Specifically, they used 3 fitness trackers for 30 minutes before they compared them and participated in a design session for designing a fitness tracker. Finally, both studies agreed that the values that people in wheelchairs may want to see are energy burned (calories), distance, GPS data, and pulse. The participants in the study by Malu and Findlater [5] added that calorie intake is equally important to energy burned. Finally, Malu and Findlater [5], unlike Carrington et al [6], showed that commercial self-tracking devices using GPS and comparing mobility in steps between different dates were acceptable and of value to their participants.

In 2019, Helle and Rosenbeck Gøegb [39] conducted a focus group interview study with 7 wheelchair users who were members of a basketball team. The study aimed to explore wheelchair users’ experiences with current self-tracking devices and future requirements. Their key results showed that their participants only found the distance and time parameters useful and perceived calorie consumption, pulse, steps, and training intensity as inaccurate [39]. Their study participants commented that “some activity trackers provide reminders that assume that the user can walk” [39]. The authors concluded that accurate activity trackers designed for wheelchair users are needed. It is not obvious from this publication whether the participants were daily users of the technology or whether the technology was given to them for study purposes, and we do not know whether they were professional athletes.

To summarize, research on self-tracking and self-tracking technology and its use by wheelchair users with SCI is primarily focused on either building technology for them or gathering requirements and their opinions on contemporary commercial self-tracking technology, with which they may have limited experience [5-7,11,25,39]. Our research enriches this body of knowledge [5-7,11,25,39] with lived experience of people who track or tracked their activity or use or used commercial self-tracking technology. This study also increases the body of knowledge of everyday lived experiences with self-tracking devices and self-tracking for an often underrepresented community in technological research—wheelchair users with SCI [40].

### More Reasons for Inclusion in the Design of Commercial Technology

People with disabilities are often considered users of “special” products designed explicitly for them instead of active participants in the mainstream market [41]. “Special” targeted products would have a smaller share in the market, and they may be more expensive and, therefore, inaccessible for many people with disabilities as people with disabilities—at least in the European Union (EU) [42]—tend to be at a higher risk of financial challenges. According to Eskyte [41], dividing the market into “average” and “vulnerable” users is an obstacle to incorporating accessibility requirements into general consumer product development. In 2025, all digital services and products in the EU market need to be accessible according to the EAA [10], which will urge practitioners to design digital products and services with a diverse audience in mind.

Publications in technological fields support that building things for people with disabilities often means building better things for everyone [9], and a systematic literature review [11] shows that research on technology for people with SCI tends toward user-centered design. Even though user-centered design focuses on the user by collecting information about them, building empathy—often by simulating their health conditions [43]—and potentially involving them in key stages of the design and development process, it does not seem to be enough. Bennett and Rosner [44] argued for a more participatory way of designing; instead of designing for the person who is different from the designers (eg, wheelchair users), we should design *with* them as part of the design team. Their research indicates that, by trying to simulate their life experiences, we do not treat them as equals but as something different from us, different from the norm [44]. While the solution to the limitations of current empathy-building methods seems to be the participation of the specific user group in the design process, participation does not guarantee technology acceptance [26] and may be hard to achieve [40]. For example, in the case of SCI, Kabir et al [40] support that, due to the impact of the condition on the user, for example, speaking or fatigue issues may have been excluded from the research because the collection methods of the designers and researchers are inaccessible. Kabir et al [40] describe that the methods need to be adjusted based on each participant’s abilities, something that some publication forums and reviewers may consider a methodological weakness of a study.

This paper does not argue about replacing the involvement of people with SCI in research or user research. However, it gives an idea of how current people with SCI experience commercial self-tracking technology and describes some of their needs. Even though researchers and practitioners will need to involve people with SCI when designing accessible products, this study can act as a starting point to understand some people’s experiences with SCI, their self-tracking habits, and their use of self-tracking technology.

## Methods

We will first present the research context and then how the data collection and analysis were conducted.

### Context of the Study

This research was conducted as part of a long-lasting cooperation among researchers in the human-computer interaction field; a researcher in physiotherapy focusing on wheelchair users with SCI; and a center for SCI in Gothenburg, Sweden. This cooperation aims to explore the use of affordable commercial wearables and build an application that uses the wearables’ data to accurately calculate the collected variables specifically for wheelchair users with SCI. The project is based on research exploring the energy outtake for wheelchair users with SCI on specific exercises [21-24]. Within the scope of this project, we conducted interviews to understand *how wheelchair users with SCI experience self-tracking and the use of commercial self-tracking technology in their everyday lives*.

### Ethical Considerations

At the start of the cooperation, the planned research was reviewed and evaluated for its ethical integrity following the process of the Department of Applied IT at the University of Gothenburg, Sweden. This qualitative study interviewed people with SCI on their tracking habits and use of commercial self-tracking technology. This type of research does not fall under any of the categories described by the Riksdag (the supreme decision-making body of the Kingdom of Sweden) as research requiring ethical clearance as no sensitive personal data or other personal data were collected or published [45]. The study did not have access to or collect interviewees' personal information prior to, during, or after interviews. The participants were informed about the study through an informed consent form and could ask questions before taking part. The information sheet also included details on how they could withdraw. The data of the participants were handled according to the Swedish implementation of the European General Data Protection Regulation. Participants received no compensation.

### Recruitment, Data Collection, and Data Analysis

We conducted 9 semistructured interviews [46,47] with wheelchair users with SCI. Knowing from the literature [40] that we may face challenges in recruiting participants for interviews, we used nonprobability convenience sampling using the network of the SCI center in Gothenburg, Sweden, and personal connections. Eligible for participation were people aged >18 years who had SCI for more than a year and who used a wheelchair to move. If participants did not fulfill all the aforementioned criteria, they were excluded. The aim was to have a deep understanding of the experiences of the people who contacted us rather than having a certain quantity of data.

Due to the geographical distribution of the participants and to accommodate the interviews at any time the participants wanted (even if it was on short notice), we conducted the interviews and recorded them over Zoom video call (Zoom Video Communications). In that way, the participants could also show us how they tracked themselves if needed. The interview guide had three groups of questions: (1) introductory and background questions, aiming to get to know the interviewees and their habits concerning daily life and exercise activity, including mobility; (2) questions about experiences of self-tracking and self-tracking technologies before and after their injury, aiming to understand the relationship of the interviewee with self-tracking and self-tracking technologies before and after their injury; and (3) closure of the interview questions, aiming to allow the interviewees to add anything they wanted us to know and understand their interest in the results of the study. We did not collect any actual measurements of their physical activity, nor did we try to evaluate their physical activity based on any tool as the focus of this study was to understand their experiences with self-tracking and self-tracking technologies.

The average duration of the interviews was 45 (SD 12.9) minutes, with the shortest being 30 minutes long and the longest being 69 minutes long (390 min; 6.5 h of video recordings in total). All participants were Swedish; 89% (8/9) of the interviews were conducted in English, and 11% (1/9) were conducted in Swedish. The participants interviewed in English could communicate clearly and fluently in English. The interviews were transcribed, and the first author analyzed them through inductive thematic analysis [48] following the steps by Braun and Clarke [49]. The first author familiarized themselves with the data and found codes and initial themes (steps 1-3). All authors reviewed, defined, and named the themes (steps 4-5). In our qualitative and explorative study, we focused on providing a rich description of our participants’ experiences with self-tracking and self-tracking technology rather than the frequency with which they talked about each theme. The quotes used were modified to remove repetitions and for grammatical correctness. The quotes taken from the Swedish-language interview were translated into English.

### Methodological Limitations

To invite the participants, we used the connections mentioned previously. In the invitation, we informed participants that it would be an online interview; thus, participants who had difficulty speaking due to their injury or other reasons may have been excluded [40]. We expected a small sample who would have the time and energy to participate. To counteract this, we invited people with SCI to participate regardless of whether they were current or past users of self-tracking technology as long as they had tracked something related to their injury or physical activity at some point. We focused on analyzing and obtaining a deeper understanding of the experiences of the 9 people rather than on collecting more data.

## Results

### Overview

A total of 22% (2/9) of the participants were female, reflecting the gender ratio of SCI at 20% female [50]. In total, 22% (2/9) of the participants had never used self-tracking technology, but they had all tracked some variables. Apart from participant P5, no one was currently an athlete. All the participants (9/9, 100%) were well past their first year of living with SCI, characterized as phase 3 or the chronic phase [40]. No participant had any serious issues with breathing or speaking during the interview regardless of their level of injury. More details about each participant’s sex, age, years with SCI, and use of self-tracking technology, as well as the code used to refer to them in the following sections, can be found in Table 1.

**Table 1:** Demographics of the participants, including their use of self-tracking technology.

Participant code	Sex	Age group (y)	Years with SCI^a^	Use of self-tracking technology
P1	Male	50s	35	Yes
P2	Female	40s	31	Yes
P3	Male	30s	5	Yes
P4	Male	≥65	29	No
P5	Male	30s	12	Yes
P6	Male	40s	13	Yes
P7	Female	≥65	40	No
P8	Male	60s	6	Yes
P9	Male	50s	—^b^	Yes

^a^SCI: spinal cord injury.

^b^Not applicable.

In total, 78% (7/9) of the participants had extensively experienced self-tracking technology; however, this high level of engagement with technology should be considered within the context of the study. People living in Sweden use IT daily for mundane things (eg, mobile payment is common; 92% of the population) [51]. In addition, Sweden is among the 7 countries in the EU with the lowest gap in employment between people with and without disabilities, which may have allowed our participants to have enough income [52] to purchase self-tracking technologies.

**Figure 1:**
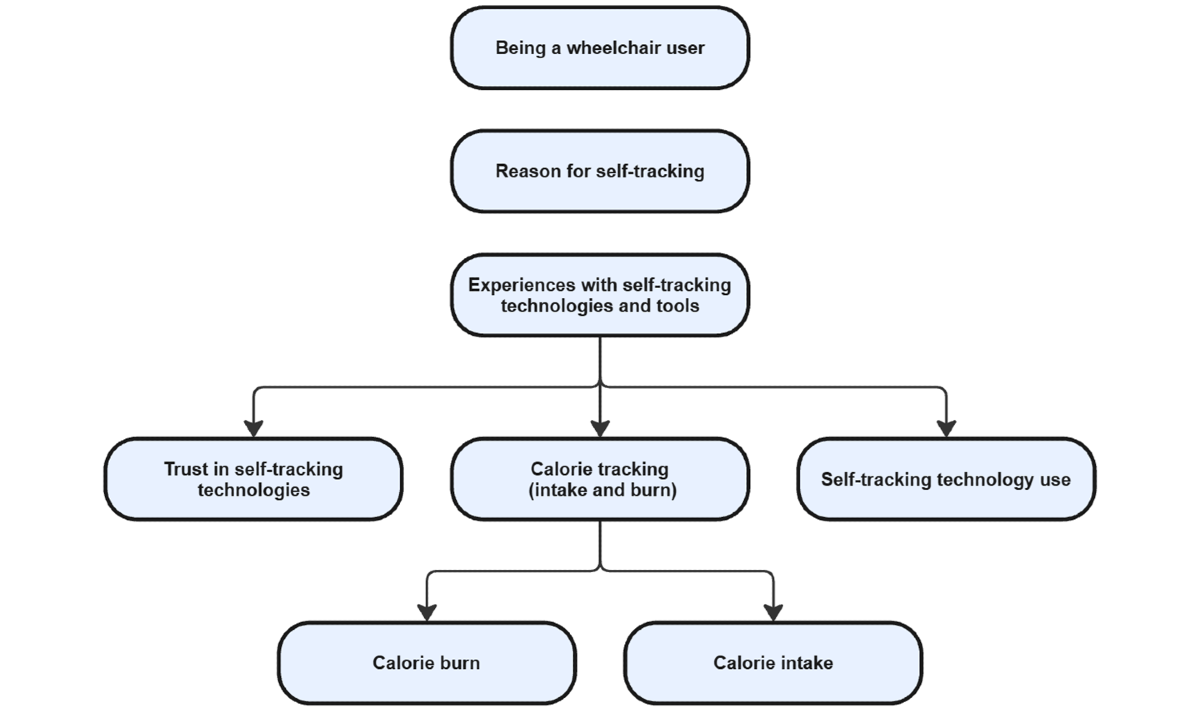
The final themes that resulted from the thematic analysis in a diagrammatic representation. Multimedia Appendix 1 shows the representation of the themes in a tabular form with a summary of the main findings.

The thematic analysis resulted in 3 themes, one of which had 3 subthemes. The first theme, *being a wheelchair user with SCI*, included codes relevant to the participants’ descriptions of their activity levels, themselves, and their lives. The second theme, *reasons for self-tracking*, included codes related to the participants’ self-tracking habits. The third theme, *experiences with self-tracking technologies and tools*, included subthemes related to self-tracking technology use, calorie tracking (intake and burn), and trust in self-tracking technologies. Figure 1 provides a more illustrative understanding of how the themes are structured. The figure is designed to be read from top to bottom and left to right. A tabular form of the figure can be found in Multimedia Appendix 1.

### Being a Wheelchair User With SCI

This presentation of the participants aims to increase our empathy and understanding of how they experience their lives as wheelchair users and as former walking persons. Wheelchair users with SCI experience life first as walking people and then as wheelchair users. This means that they must adjust to their new body and develop new skills and often see themselves and their life divided into 2 periods—before and after the injury. For example, P8, who had a physical job and changed to a desk job after becoming a wheelchair user, mentioned that “before the injury, I did not track [my activity]. You could see I was physically in a good place. I was very masculine. After the injury, it is another story...you need to train, of course, a lot of rehabilitation.” The various SCIs can influence people’s bodies and lives differently, widening the diversity of their bodies.

Our participants dealt with the change in their skills in five ways: (1) adjusting their previous knowledge to the new situation, (2) giving up old habits, (3) being challenged to keep old habits, (4) reflecting on the behavior of their body, and (5) acquiring new skills. For example, regarding item 1, P3 adjusted his previous knowledge to the new situation in the following way:

I knew what because of my experience before the injury. I knew how to train, [but] I had to adapt to the situation I got into. I could not do everything in the same way, but I got some tips from the rehabilitation clinic, and I tested them myself.

Others had advanced skills as non–wheelchair users that they lost when they became wheelchair users. For example, regarding item 2, P9 preferred to give up old habits “since I know that I have been playing badminton at a certain [high] level, it is frustrating to find myself being bad at it in the wheelchair. So maybe I would rather play tennis or basketball,” whereas others saw their new life as a challenge to keep doing what they used to do. For example, regarding item 3, P1, who used to ski before his injury, felt that sit skiing would not be a challenge. When he tried it and realized how difficult it was for him, he was challenged to keep skiing:

I tried [sit ski], then it was so super hard.... So somewhere, it triggered me that I need to practice to get better at it.

Regarding item 4, P9 reflected that his body did not react the same while running. As now he was not using his legs but his arms to move the wheelchair, it was harder for him to raise his pulse and feel the effects of exercise before his arms got tired. All of them had to learn to use a wheelchair, and the learning experience differed from person to person (item 5). For example, P4, one of the main organizers of a wheelchair community, mentioned that, when he started using a wheelchair, there was not much support but, in his community, they support others with basic wheelchair skills:

[W]e [practice] with balancing the wheelchair [on the back wheels] without dropping it back. [It] is very useful and hard skill.... It is very dangerous [as] you can hit your head.

Everyday activities take more time based on the level of the injury and how it has influenced the body. For example, P7 mentioned that “I have to take care of myself and then work...it takes a lot of time,” so she did not have much time to dedicate to exercising. P6, who worked at a rehabilitation center where people stayed for several weeks, said the following:

[R]ehabilitation is a lot about...you come here for 4, 6, 8, 10 weeks and you lay a foundation, but to get to be autonomous/independent. You need to continue when you get home because that’s where you really need to put in the effort. And I tried to be clear with patients that this is the beginning. When you come home, that’s where the struggle or the real-life starts.

P6 underlined that, when one is in the rehabilitation clinic, they are free from daily responsibilities that may increase when they return to their daily life and take time from the exercise levels they need to sustain. Finally, P4 and P8 pointed out that, in addition to SCI, one develops more chronic conditions due to aging (eg, P8 kept track of his diet due to type 2 diabetes, and P4 tracked his blood pressure and related medication because he had a myocardial infarction).

To summarize, wheelchair users with SCI need to learn to know and adjust to their new bodies—bodies that are diverse by nature combined with the type of injury. When they learn basic wheelchair skills, they must deal with everyday life, which of course takes time for everyone, but for them, it can take time from their exercise. Exercise is a vital part of the high level of activity they need to sustain to be independent and healthy.

### Reasons for Self-Tracking

Habitually, inpatients in rehabilitation clinics are told to document their activities or have them tracked during any type of rehabilitation or training related to their injury. However, few of our participants described this as tracking. For example, P9 mentioned the following:

If one is in rehab, one should keep track so they can see that the exercise is working...

P7 remembered tracking during her injury-related training:

[Y]ou get a mentor that you can check up to after a couple of weeks, then you must make a new measure and see how you are doing. That was very useful.

P8 reflected on the lack of technology and feedback during rehabilitation:

I think I get the idea [of tracking] from having to fill out all those forms when I was in the rehab center. Every week I have to write on paper, my weight, how much I lost when I went to the toilet, how much I ate on a particular day...and then the doctor concluded...[what I should change]...with the technology we have today just putting that on paper was not doing anything to me...I was just waiting for the outcome from the doctor.

Our participants had different motivations for tracking. A total of 44% (4/9) of them (P1, P2, P5, and P9) mentioned that they had started tracking as they were practicing a sport. They tracked because their coach had told them to, or their coach did the tracking for them. However, all of them kept tracking their behavior even after stopping exercising. P2 tracked variables related to swimming (eg, swimming style, time, and meters) as she was training for the Paralympics; nowadays, she tracks the activities she does through her Apple Watch as “it’s so simple...the watch does it for me” and also uses it as a motivation to move—“it kind of reminds me to move” and “it keeps track, and it tells me you did a good [job] today.” P1 mentioned his reaction the first time he was asked to track variables related to skiing (eg, weather conditions, exercise type, and timing) and how he came to keep tracking his activities:

Well, it was the trainer who said, you need to keep track on what you do. Why I’ve already done it. That was my answer. But then I started to do it. And then I realized that let’s say after one season, I looked in the diary [and thought] oh, I need a lot of those things and [those things] didn’t pay off. Maybe I should do less of those things since they don’t pay off so much and maybe I should increase the things that pay off.

P9 continued reflecting on his motivation for tracking his activities using his Apple Watch:

[I]t is not so much that I want to be in a national team or anything, it is more important that I can stay healthy and be a part of family activities and continue to do that. That is the most important thing to me.

P3 mentioned keeping track of his training, especially when making changes to his training activities:

When you gonna train intensively for a month or something, I think it’s interesting to keep track of your training. If you have a goal or race as well, which I don’t right now.

He reflected the following:

Yeah, I think most parts of tracking is for motivation for me at least, and then also, it’s just interesting to see some data.

P2 and P3 mentioned that they used tracking to increase motivation, as did P6:

[I]t’s mostly motivation to see that to give myself a pat on the shoulder.

Some participants (P1, P5, P6, and P9) tracked their activity before their injury as they were practicing different sports or participating in competitions. On the other hand, P4, P7, and P8 were not interested in tracking or were motivated to track only after their injury. P4 tracked his activity only as part of a game his colleague created—a point system that considered activities one did (eg, walking) and measurements of weight, waist, and chest. The person with the most points won. The point system was adjusted for P4 as he was in a wheelchair. P4 mentioned this game:

It was like, a challenge, you know...we challenge ourselves and even checked together “now I’m up to 2000 points” or whatever.

P7, who had a higher level of injury, mentioned that she was never interested in tracking but that she kept track of her weight and the time it took her to move between places in her house:

[I]f you go to the bathroom, I got to make it in 10 minutes, [to] compete with myself to do with it as quickly as possible.... The only thing I track is my weight. My heart rate is no use because I can’t get [much variation]

P8 mentioned that he started feeling the need to track after his injury; before, he could simply see that he was muscular.

Summarizing, our participants performed a lot of tracking during their rehabilitation period to support the health care professionals following them, but the tracking was done on paper. Those training before their injury had a habit of tracking different variables and found value in tracking, but not everyone felt the need to track. In any case, the injury changed the way in which our participants tracked, either from not needing to track to needing to track or by changing the reasons for tracking and the variables they tracked.

### Experiences With Self-Tracking Technologies and Tools

In this section, we will present the experiences of our participants with self-tracking technologies and tools through three subthemes: (1) use of self-tracking technologies, (2) tracking calories, and (3) trust in self-tracking technologies.

#### Self-Tracking Technology Use

The participants had experience using the Apple Watch, Runkeeper, Polar Beat, Pulse Polar Watch, and Nike+. While these technologies automate tracking and keep score of data, the participants also used manual means of tracking data about themselves. They mentioned using Microsoft Excel, a stopwatch, Lifesum, and paper as examples of manual means of self-tracking. One participant (P5) also mentioned that they “track in my mind.” However, the most recurrent technologies in the responses were Apple Watch and Runkeeper.

Before their injury, only P6 and P9 used technology (Nike+ Running App) to track their activity. P1 kept a logbook with ski-related variables (he continued after the injury until he bought his Apple Watch), and P5’s coach tracked his activity and gave him a schedule or advice accordingly. After their injury, participants P3, P5, P6, and P9 started using the Runkeeper app, which includes a wheelchair user mode. P3 and P5 used it to measure their speed and the distance they ran, and P6 and P9 used it to train for marathons or similar events. However, P9 mentioned that he had given up on Runkeeper because it did not work well with his Apple Watch:

Nike, that’s actually what I used when [training for the] world run because it’s, it’s pre-installed in my Apple watch. So, when I got the Apple Watch, the first thing I did was download RunKeeper on my Apple watch. But I noticed that it wasn’t attached to it with all its [features]. But there was also already a running up called Nike.

P1, P2, and P9 were using Apple Watch to track their activities. Apple Watch includes an option for wheelchair users. In the default mode, the Apple Watch tracks many activities (eg, biking, swimming, and skiing). However, if one sets it to the wheelchair user mode, only 4 activities become available: wheeling in- or outdoors and walking or running speed. P2 preferred to track her activities separately and used the default mode:

So most of the time I use the activities that are in the watch. I’m aware that they [calories] are not adapted for me and my muscle mass. That is not the important thing to me. For me, the important thing is that kind of keeps track and tells me that—okay, you did a good job.

For P2, it was more important to track her activities and obtain positive feedback than to have access to accurate measurements of some of her activities.

The Apple Watch also displays 3 concentric rings: the inner blue ring shows how many times in the day one has moved (ie, changed position), the middle green circle shows how many minutes of brisk activity one has had, and the outer red circle indicates the active calories burned throughout the day. When P2 referred to the positive feedback she received, she meant how full these rings were. P1 and P9—who used the wheelchair mode on their watches—also looked at those rings for a quick daily overview. P9 mentioned the following:

[I] look at it but I don’t pay too much attention to it.... I can see that I have done my full day’s work and that’s kind of the analysis I do, not so much else.

Some of our participants used other apps and devices. For example, P8 had tried the Samsung Health app, but the measurements provided were not relevant to him. P1, one of the Apple Watch users, used a digital food diary (Lifesum) compatible with his watch to track his diet. P5, who was using Runkeeper, also used Polar Beat when training to measure his heart rate; however, he also considered the limitations of the technology. Specifically, he said the following:

I don’t know if it’s correct, but they mean that if you’re a tetraplegic [injury level], your heart rate can’t go over 120 Mm. And you can push as hard as you can. And you can’t get over 120 But on the Polar-Beat that I [wear around the arm], I have been up to 136, I don’t trust the one you put around [the chest] like this.

This section shows that our participants were familiar with self-tracking tools. Many used or tried digital self-tracking technology after their injury, and some had done it even before. They tracked variables that could influence their sports performance or support them in understanding their body (eg, pulse and diet). They also tracked in less quantifiable ways, for example, what activities they did (eg, skiing or swimming) or the completion of goals set on their Apple Watch, which provided motivation. However, all participants mentioned calories and their measurement and consumption. Some expressed the need for a “lifestyle app” supporting them in healthy living as a whole—both in activity and diet tracking.

#### Tracking the Calories

##### Overview

When our participants talked about their experiences with self-tracking technology, the discussion always turned to calorie tracking or similar variables (such as weight and diet). Keeping a healthy lifestyle and not gaining weight is an issue for wheelchair users, and the impact of gaining weight on their life quality is tremendous. P9 was the most descriptive on how gaining weight could influence his life quality:

I just want one thing; I don’t want to have to change my wheelchair into a wider one. If I get fatter, for example, it would be harder to make all these movements that I have to do in and out of the car every day, in and out of my bed every day in and out of the shower every day, or maybe every other day. Also, if because I’m getting fatter, if I have to get a wider wheelchair, I wouldn’t get into certain doors, for example, in hotel rooms because my wheels are wider apart...I don’t want that to happen and I’m ready to try any mechanical device to help me from not having to do that.

This quote illustrates the potentially more serious consequences of gaining weight for wheelchair users and the need to track the calorie burn and, if possible, intake. P5 mentioned that “in our world, calories are very important,” and P1 commented on weight gain and aging:

[W]eight is a problem all the time, especially now when I am getting a bit older, and it is easier to gain weight than losing it.

Even P7, who seemed uninterested in tracking her activities and did not use any self-tracking technology, mentioned that “tracking it’s not so important for me” but “I track weight because it is important for us.” When our participants discussed tracking their calories, they mostly talked about calorie burn (P1, P2, P3, P5, P6, P8, and P9). Only 44% (4/9) of the participants (P1, P3, P4, and P5) mentioned calorie intake or diet, and only 11% (1/9; P7) mentioned weight (which can be perceived as a balance between calorie intake and burn).

##### Calorie Burn

As wheelchair users with SCI have less muscle mass than a walking person, they consume calories differently. P1 mentioned the following:

It is really hard for a paraplegic [injury level] to burn calories. It takes so much more time to burn the same amount as you [a full-muscle ability person]. If you’re out jogging for one hour, I must wheel maybe for three.

Therefore, to burn enough calories, they need to spend more time exercising.

Few commercial self-tracking technologies have their features adjusted to wheelchair users. Runkeeper and Apple Watch were 2 technologies mentioned by our participants that consider wheelchair users in some of their features. Our participants did not trust the self-tracking technology they used regarding calories and, instead, used other variables as a proxy for calorie burn. Consequently, they managed their calories by staying active or using self-tracking technology as a motivation to stay active. P6 used self-tracking technology (FitNotes and Runkeeper) to keep a high level of activity in addition to motivating himself:

[One session] wasn’t good enough, next week, I’m doing two sessions. And I’m taking the time [for an extra session] by putting something else out of my schedule. Or if, last week, I trained five times. I tell myself I did something good. Continue!

P5, who used the Runkeeper app, underlined that he checked the distance and speed provided by the app but not the calories despite mentioning that calories were very important to him. He also discarded the calories that his wearable showed (Polar Beat, which measures heartbeat and calories), saying that “I don’t care about the calories because I know...the calories are all wrong.” P3 also mentioned heartbeat as an indicator for understanding that his training had an impact:

[H]eartbeat is always good because if your heartbeat races, you're doing something.

He also reflected on a noncommercial smartwatch he had tried as part of another study that measured steps, finding steps not to be a good indicator or proxy for understanding his activity levels:

I do not know if I am moving a lot, I have more steps on my watch, but I do not know how it works and I do not know what it reduces it and what it does not.

Similarly, P8 reflected that the visualization of steps in commercial self-tracking technology as an activity proxy was irrelevant to him:

Samsung health (smartphone) counts my steps which is not connected with the reality...Like FitBit.

Apple Watch is one of the self-tracking technologies that has adjusted some of its features for wheelchair users (eg, wheeling in- or outdoors and walking or running pace); however, only P1 trusted and followed their calorie burn. In contrast, P9 pointed out the following:

I have my Apple Watch on when I do [strength exercises]. I guess it’s not smart...it tracks some of the movements I make as a push in my wheelchair. It was the same with that would that pole pooling...it doesn’t track it as something else than kind of push with the wheelchair.

Thus, the watch counted his calories based on the strokes he did to move his wheelchair, and he argued that, regardless of what exercise he did, the watch would always measure only wheeling in- or outdoors and walking or running pace but not the actual exercise. Therefore, the calories could not be estimated based on a different activity but only on the strokes he did, so he did not pay attention to them but used the watch mainly for motivation. P2, another Apple Watch user, said the following:

[A]ll other activities are in there, but they are not adapted for people with no full muscle ability. With that said, I had kind of decided that it doesn’t really matter if I spent 300 calories or 500 calories.

She added that she used the watch to motivate herself to move. Thus, she preferred to use the Apple Watch in the default mode rather than in the wheelchair mode, favoring the ability to track different activities over a more accurate calorie estimation.

Some participants also used nontechnological means to measure their calories manually. For example, P1, who was part of a medical research project on SCI, became aware of the connection between his energy consumption and his pulse and took action based on that:

[B]efore being part of the project about energy consumption for a disabled person, I had no way to determine how much energy I consume during the day but after that, I made charts because I have a really strong correlation between pulse and how much energy I consume.

To summarize, a person with SCI consumes calories at a slower pace than a full-muscle, able-bodied person and needs to be more active to burn the same amount of calories. Most commercial technology currently in the market does not account for this difference, which leads to apps incorporating the wheelchair symbol without adjusting the calorie burn for this type of user or apps calculating the calorie burn correctly but only for limited activities. Thus, our participants estimated their calorie burn based on how many exercise sessions they did or their heart rate increase. Self-tracking technology was used mainly for motivation, and steps were not perceived as a good proxy for activity tracking or calorie burn.

##### Calorie Intake

Weight is impacted not only by calorie expenditure but also by calorie intake. For example, P4 said that “Exercises are good but if you don’t stop eating too much, you will never lose weight.” Just like P3 adjusted his exercise knowledge to the new situation, P5 needed to adjust what he knew about diet. Before the injury, P5 had a personal trainer who planned his weekly exercise and diet, for example, to gain muscle. However, P5 mentioned that he found that he could no longer follow what he had learned from his trainer after the injury:

When I ended up in a wheelchair, I was used to having the [diet and exercise] paper in the back of my head. But every other person in a wheelchair said you can’t eat as much as you did before. So today I’m eating small portions but more often. I don’t do breakfast, lunch, dinner.

P5 had to adapt his weight management after becoming a wheelchair user by avoiding the traditional food distribution throughout the day.

Even though participants were interested in counting their calorie intake, only P1 used a dietary app, called Lifesum. He mentioned that the intake of calories was also important and that it would be interesting to have a daily calorie goal and take note of the type of diet one follows, if applicable. P1 said the following:

An app that could measure what I eat, and I can put in parameters like, [amount of] calories [that] are ok for the day, and any kind of diet I follow.

Another participant, P7, felt that she had control over her diet owing to a course organized for people with SCI that she took after her injury:

[In this course] you start tracking everything, they check your weight and height, how you train and exercise, how you feel—everything. And then you get a plan and learn a lot [about] bad calories, and you know exactly how much you are going to eat and how to change...you get a mentor that you can check in with after a couple of weeks, then you [must] make a new measure or everything and see how you are doing. That was very useful.

Here, instead of using a tracking tool, the user learned how to manage their calorie intake in an educational setting, when episodic measurements were made, and then made sense of them with the support of their mentor.

The participants expressed that calorie intake was as important as calorie burn. They described that they had to adjust their eating habits after the injury to fit their new bodies. Even though only P1 used self-tracking technology to track their diet, other participants mentioned that they had to undergo training or go through an adjustment period to learn how much they could eat by tracking their diet.

#### Trust in Self-Tracking Technology

P1, P2, P3, P5, P6, and P9 used self-tracking technology to track their activities but did not trust this technology, especially regarding the calories. P1 trusted his Apple Watch because “Apple bought a survey conducted in the US that was about paraplegics, working [out], and how much calories [they burn] with which workload. And they put that algorithm in the watch. For example, let’s say a paraplegic person has a 30% slower or lower metabolism compared to able-bodied, that is in consideration in this app.” He compared his watch to Runkeeper:

I’m out wheeling with 100 pulses; I get depressed because it’s like 210 calories. But if you go to RunKeeper, and you have been jogging for one hour, you have 700 calories. So, if you use that app, you will be so fooled and tricked by it. Because it is hard for a paraplegic to burn calories.

P1 made a comment on commercial apps using the wheelchair symbol deceptively:

[Y]ou have apps on the market RunKeeper, or whatever, you download it because it has a wheelchair symbol [thinking] Oh, yeah, this is a cool company, they even bother to [consider] wheelchair [users]. Yeah, but people don’t understand. That’s a chart with calories from an able-bodied person. They just replace the running person symbol with the wheelchair symbol.

P2 was dissatisfied that, as a wheelchair user, she could only track 4 activities on the Apple Watch. Therefore, she preferred to use the watch without the wheelchair user setting to keep track of the different activities rather than accurately tracking the calories—as the quote in the previous theme shows. Despite this, P2 stated that they were an “Apple family” and that she trusted Apple because “it’s people I know that are interested in technology that buy all the gear and try all the gear out that’s Apple Watch is good I’m buying kind of trusted them Yeah. And my husband recommended and said that it would be worth it [now] that I had already started to add more exercise into my life, and I thought that could be a good fun thing to try.” In this case, what made the technology more trustworthy was not the actual technological features but the social context. On the other hand, P9 did not trust his Apple Watch to accurately calculate the calories because it tracked everything as strokes on a wheelchair regardless of the type of activity—as his quote in the previous theme showed. However, he mentioned that he trusted his Apple Watch more than an app on his phone as he could not see how an app could track accurately:

[T]here was a wheelchair feature that I can put [in the RunKeeper], instead of running or cycling, I put it on the chair. But for example, it didn’t track my pushes, because it can’t track my pushes, it seems it’s only on the phone. So, I think it helps that it’s on my Apple watch on my hand to be able to keep track of [the pushes]. So, if I only have the phone in my, pouch that I have on my sitting pillow, or I have it on my arm higher, it doesn’t track my movement in the same way.

P9 expressed his distrust of Runkeeper because it did not provide the user with an explanation of how the app measures the calories of a wheelchair user.

Similarly, P3 and P5 mentioned that they did not trust the technology they used (Runkeeper and Polar Beat) regarding the calories it presented them with. Specifically, P5 mentioned the following:

Now it’s like, if I go for a walk, it shows how far and the pace and even it [RunKeeper] shows the calories. But I do not think the calories are exact, so I don’t care so much about the calories.

He explained that, apart from calories, “I trust just the RunKeeper because it’s like a map, a GPS. You have walked three kilometers. You got the pace for each kilometer*.*” This quote illustrates that, despite P5 distrusting some features of the app (calories), he trusted others that were more easily understandable. He added the following:

So for now, I’m pretty happy with the Polar-Beat it is just the calories are not...and it’s not reliable.

Although the user considered himself satisfied with the Polar Beat use, he chose not to rely on the calorie measurement.

Summarizing, trust in self-tracking technology (or the lack of it) is influenced by the perception of the participants regarding how the technology works and how the development company accounts for different bodies related to calorie burn.

## Discussion

### Principal Findings

This section recaps our contribution followed by a short discussion on trust and transparency related to self-tracking technology. Then follows a section with the implications of commercial self-tracking technology, which concludes with directions for future research.

To address the question of how wheelchair users with SCI experience self-tracking and commercial self-tracking technology in their everyday lives, we interviewed 9 wheelchair users with SCI who had experience with self-tracking or self-tracking technology. The last theme, *experiences with self-tracking technology and tools* in Figure 1 divided into 3 subthemes, shows that current self-tracking technology fails to accommodate wheelchair users’ needs concerning calorie management in 2 ways. First, the current commercial technology calorie calculation is based on a body with full muscle ability and, therefore, is inaccurate for people with SCI (subtheme: *calorie tracking*), and second, the users do not trust the calories in the self-tracking technology (subtheme: *trust in self-tracking technology*). Nevertheless, the wheelchair users we studied continue to use self-tracking technology to motivate them to stay active and to monitor variables such as heartbeat, speed, and activity schedule as proxies for their energy consumption (subtheme: *self-tracking technology use*).

This research has limitations due to the small number of interviews and the positioning of the research in a technologically advanced country (Sweden), which also has 1 of the 7 lowest gaps in unemployment between people with and without disabilities in the EU. If the study had been conducted in a different context with, for example, lower technological literacy and use or lower employability of people with disabilities, the results may have differed, particularly on the number of people who have used self-tracking technology. Finally, all of our participants (9/9, 100%) were White. If our participants had had a darker skin tone, the results may have differed, particularly in relation to the *trust in self-tracking technology* theme as self-tracking technology is even less accurate on darker skin [53]. Nevertheless, this study adds to the body of knowledge by validating previous studies on the different variables that wheelchair users want to see. It expands the current body of knowledge by focusing on long-lived experiences of self-tracking and contemporary commercial self-tracking technology, especially on calorie management—the most important factor influencing the life quality of wheelchair users with SCI.

Our research contributes to the scientific community as follows. It extends the work by Malu and Findlater [5] on fitness trackers on wheelchairs by interviewing wheelchair users with SCI who had lived, daily experience with tracking their activity or self-tracking technologies. In line with the work by Malu and Findlater [5], our findings in the *calorie tracking* subtheme indicate that one of the most important values to be measured is calories (intake and burn), followed by distances, GPS data, and pulse, as indicated by the *self-tracking technology use* subtheme. However, our findings in the *calorie burn* subtheme indicate that steps are not a good variable to measure for wheelchair users, which contradicts the study by Malu and Findlater [5] but is in line with the work by Carrington et al [6] and Helle and Rosenbeck Gøegb [39].

Our research, specifically the *self-tracking technology use* subtheme, is in line with the work by Helle Rosenbeck Gøegb[39] regarding the tracking of distance and time. In addition, the *trust in self-tracking technologies* subtheme is partially in line with the results of Helle and Rosenbeck Gøegb [39] showing a lack of trust in technology related to some variables (calorie burn and training intensity). Our participants, similarly to those in the study by Helle and Rosenbeck Gøegb [39], trusted the self-tracking technology regarding their speed measurements and were interested in these measurements; however, neither the participants in the aforementioned study nor our participants trusted calorie burn tracking (see the *trust in self-tracking technology* subtheme). Some of our participants showed a lack of trust in training intensity as well (similarly to those in the study by Helle and Rosenbeck Gøegb [39]); for example, smartwatches counted every activity as a stroke on a chair without distinguishing it from upper-body strength training (see the *trust in self-tracking technology* subtheme). Finally, our findings in the *reasons for self-tracking* theme suggest that being able to track different activities and calorie burn and keep track of exercise schedules, as well as seeing activity goals being reached, was motivational for our participants to keep the active lifestyle they needed, which is in line with the findings of past research [27,28,54].

### Trust and Transparency

Participants’ trust in self-tracking technologies was heavily influenced by their understanding and perception of how these devices worked. For example, Apple Watch users often trusted the device more than phone apps, believing that the watch’s ability to track arm movements made it inherently more accurate than a phone carried in a pocket. However, this trust was conditional and limited; participants expressed confidence in the calorie estimates only for the 4 wheelchair-specific activities provided by the device. Regarding other activities in which calorie expenditure was approximated using these predefined categories, they expressed skepticism about accuracy. This led to varying strategies—some participants attempted to fit all their activities into 1 of the 4 wheelchair-specific categories to achieve more reliable calorie estimates, whereas others discarded calorie tracking entirely and used the watch in nonwheelchair mode to access a broader range of features.

Previous work on trust in self-tracking technology has revealed that users often have a *mental model* of how apps or devices work and calculate data [55]. Trust in self-tracking technologies, particularly in the context of wheelchair users with SCI, hinges significantly on the transparency of how data are collected, processed, and presented. Participants in our study exhibited a nuanced understanding of their devices, often forming mental models to rationalize the data’s validity. However, the perceived “black box” nature of calorie calculations—whether on the Apple Watch or Runkeeper—fostered skepticism. This reinforces previous findings that transparency regarding algorithms and data processing is critical for trust [56]. On the basis of this, we suggest that designers prioritize user education by including detailed explanations of their algorithms, especially when adapting generic metrics such as calorie burn for niche user groups, such as wheelchair users. This aligns with broader calls [57,58] for designing technologies that foster informed trust.

Furthermore, trust in technology is not isolated from social factors [59]. Apple Watch users’ trust was partially rooted in social recommendations and previous brand experiences. This suggests that trust may extend beyond technical accuracy to include the brand’s perceived reliability and commitment to inclusion. Investigating the interplay between social networks, brand trust, and technology adoption among wheelchair users could provide further insights into how collective validation influences individual trust decisions.

Distrust in calorie tracking was not merely technical but also emotional, linked to fears of inaccuracy impacting critical life decisions (eg, managing weight to maintain mobility). This illustrates that trust is not just a cognitive process but a deeply affective one. Incorporating participatory design with wheelchair users at every stage of the design and development process—testing, iteration, and after launch—can mitigate such emotional concerns. By co-designing solutions, developers can address both the technical and psychological dimensions of trust.

We observed how distrust often led participants to bypass calorie metrics altogether, choosing instead to focus on other variables or manual methods. This self-empowerment reflects resilience but also signals a failure of the technology to deliver on its promise. Therefore, tools must enhance user agency by aligning design goals with real-world constraints and expectations.

Finally, we observed a mismatch between representation and actual design. Runkeeper users appreciated the app’s reliance on GPS and speed data, which they found straightforward and comprehensible. However, despite this, they distrusted the app’s calorie calculations even when using its wheelchair mode and described feeling misled by its implied inclusivity. The use of wheelchair symbols created an initial perception of inclusivity but, ultimately, resulted in feelings of deception among participants due to inaccurate calorie calculations. This reflects a broader issue in which symbols and icons signaling accessibility and inclusiveness are in fact merely superficial. Misrepresentations such as these can diminish trust but also risk alienating the very communities that the technologies claim to support. Implications such as these are discussed further in the next section.

### Implications for Commercial Self-Tracking Technology for Wheelchair Users With SCI

The most important need and valuable variable for our participants (subtheme: calorie tracking), and of the participants of previous research with wheelchair users, was the accuracy of calorie consumption [6,39,60]. Previous research [6,39,60] and our participants indicated that commercial fitness and wellness technology that uses the wheelchair symbol does not necessarily calculate accurately the calories burned by wheelchair users (subtheme: *trust in self-tracking technologies*). Research [6,39,60] also indicates that specific technology for wheelchair users needs to be developed, personalized, and tailored to their needs, which underlines the importance of tailoring mainstream self-tracking technology to the user group that it intends to include [61]. Furthermore, there is extensive research on wheelchair user–specific technology for self-tracking health and well-being [27,28,54], as well as exploring current technology with wheelchair users to inspire designs of wheelchair user–specific fitness and wellness technology [5]. Even though a big part of the aforementioned literature focuses on assistive technology or technology specifically for wheelchair users, mainstream self-tracking technology that aims to be inclusive toward wheelchair users can take advantage of what assistive technology research can offer (eg, databases and artificial intelligence models) [30,31], as well as what the medical field offers, which already has some physical activity guidelines for wheelchair users [14,32,34,62].

On the other hand, the production of new wheelchair-specific self-tracking technology (which is not assistive technology) divides the market into wheelchair users and non–wheelchair users. Recent research [41] indicates that, by excluding specific populations from the development of commercial products, the incorporation of accessibility requirements into general consumer products comes to a halt. In addition, special technology could cost more as it targets a specific population. Regarding wheelchair users, special technology may not be a financially viable solution as they have a higher risk of financial issues [42]. Working with affordable materials to create new technology for a specific population can also lead to them not using it because it could be stigmatizing [63]. From 2025 onward, digital services and products in the EU market should be accessible according to the EAA [10,49]. Our findings show that the self-tracking technology currently in the market is not prepared to be accessible to wheelchair users with SCI. This technology is not trusted when it comes to calorie calculation. Our participants described how they used proxy variables to estimate their calorie consumption and activity level. They expressed the need for a self-tracking technology that accurately calculates their calorie intake and burn during a variety of activities. Many of the issues with self-tracking technology that uses the wheelchair symbol can be solved by involving wheelchair users in the design and development phase. This involvement will make the technology more relevant to the people it aims to include (ie, wheelchair users) and open the market to broader audiences [64-66].

On the basis of the literature presented in this paper and our findings, we see a need for future research and development regarding inclusive self-tracking technology for wheelchair users with SCI supported and inspired by assistive technology literature.

First, there is a need to explore ways to make affordable technology already used by wheelchair users closer to their needs and make it more accurate regarding calorie burn (ie, track a variety of activities and calorie intake). Our study can be seen as a starting point for this exploration of commercial fitness self-tracking technology and its use by wheelchair users with SCI, complementing previous literature on similar subjects [5-7,11].

Second, we argue that there is a need to confront and acknowledge the limitations of the methods used to conduct similar research and alternate them for the research and practice to be more inclusive. According to Kabir et al [40], data collection and design methods may be inaccessible to some populations, leading researchers to exclude them as they perceive them as “difficult to reach and research.” Instead, they suggest having a palette of methods and adjusting them based on the participants’ abilities [40]. Similarly, Moon et al [37] provide a comprehensive toolbox for more inclusive user research when designing with wheelchair users in mind. In addition, Li et al [30] and Huang et al [31] provide freely available concrete tools (ie, databases and code) for developing an inclusive self-tracking technology to be used by anyone wanting to make self-tracking technology more inclusive.

Third, regardless of all the support that this paper and the literature provide, the inclusion of wheelchair users as an integral part of the design process of the self-tracking technology targeting this demographic should be seen as mandatory for the product to be relevant to the new audience (ie, wheelchair users) [65,66].

### Future Research

Our next steps are a series of participatory workshops in which we—together with people with SCI and the support of the SCI center we partnered with—identify further tracking needs and co-design a first version of an app that could support them in learning about their new body in different stages of their postinjury phase. In the workshops, we plan to include the physiotherapist who cooperates with us to include the health care perspective without compromising the power balance. His participation will also create an environment in which the participants can learn more about SCI and how the health care system perceives SCI and them as patients. To create a link for the communication between health care professionals and people with SCI, we will use the connections of our partner at Karolinska Institute to investigate the needs of health care professionals (ie, which of the data tracked by people with SCI are relevant to the health care professionals and in what form to fit their practice when consulting with their patients).

## Appendix

Multimedia Appendix 1 The final themes resulting from the thematic analysis in a tabular form.

## Abbreviations

EAA: European Accessibility Act
EU: European Union
SCI: spinal cord injury
