# Supplementary material for: Experiences of Wheelchair Users With Spinal Cord Injury With Self-Tracking and Commercial Self-Tracking Technology (“In Our World, Calories Are Very Important”): Qualitative Interview Study
Source: JMIR Hum Factors. 2025 Apr 15;12:e65207. doi: 10.2196/65207 (PMC12041821; doi:10.2196/65207)
Supplement: Multimedia Appendix 1 [file humanfactors_v12i1e65207_app1.pdf]

Multimedia Appendix 1: The final themes resulting from the thematic analysis in a tabular form

Themes	Sub-themes	Sub-sub Thems	Main findings
Being a wheelchair-user			Reaction to the change of their skills after the injury (1) adjusting their past knowledge to the new situation, (2) giving up old habits, (3) being challenged to keep old habits, (4) reflecting on the behavior of their body, and (5) acquiring new skills.
			Everyday activities take longer as SCI-related exercises add to work and personal life.
			Aging and comorbidities in addition to SCI
Reasons for self-tracking			(1) Participation in sports (e.g. training, diet, sports variables (2) getting to know the body with SCI (e.g. rehabilitation) (3) Monitor activity levels<br>(4) Motivation to keep active (5) Gamification, competition, and goal setting
Experiences with Self-tracking tech. and tools	Self-tracking tech. usage		Technology and tools used for tracking: Apple Watch, RunKeeper, Polar-Beat, and corresponding sensors, Pulse Polar Watch and Nike+, Excel, a stopwatch, LifeSum, and paper
			Variables tracked: Speed and distance, different activities, activity levels/exercise sessions, heart rate, and diet
	Calorie tracking	Calorie burn	Important variable but calculated only through proxies e.g. heartbeat, high level of activity (exercise sessions/week) – steps are not a good proxy for calorie burn
		Calory intake	Adjust eating schedule, and portions,<br>Change eating habits
	Trust in Self-tracking tech.		Skeptical about the accuracy of calory calculation mistrust of wheelchair symbol in self-tracking technologies
			Trust in calorie measurements increases when (1) explicitly know that the calories are calculated with wheelchair users in mind (2) they understand how the technology works
			Trust the firm without trusting the calories
